# Residual Pulmonary Vascular Obstruction Following Mechanical Thrombectomy for Submassive Pulmonary Embolism: A Single-Center Analysis

**DOI:** 10.1016/j.jscai.2023.101260

**Published:** 2023-12-19

**Authors:** Brian Stegman, Anirudh Kumar, Thom Dahle, Wade Schmidt, Jacob Dutcher, Tanya Glenz, Daniel Appelbaum

**Affiliations:** a CentraCare Heart & Vascular Center, St. Cloud, Minnesota; b Northwestern Medicine Central DuPage Hospital, Winfield, Illinois; c Biological Sciences Division, University of Chicago Medicine, Chicago, Illinois

**Keywords:** mechanical thrombectomy, pulmonary embolism, residual pulmonary vascular obstruction

## Abstract

**Background:**

Residual pulmonary vascular obstruction (RPVO) following pulmonary embolism (PE) is associated with residual dyspnea, recurrent venous thromboembolism, and chronic thromboembolic pulmonary hypertension. Historically, acute PE treated with anticoagulation alone results in high rates of significant RPVO. Contemporary treatment of submassive PE often involves catheter-based interventions, including mechanical thrombectomy (MT), although their relation to RPVO is not characterized. In this study, we aimed to evaluate the rate of ≥10% RPVO in patients treated with MT.

**Methods:**

Twenty consecutive patients with submassive PE in a single center underwent MT and subsequent planar ventilation/perfusion scintigraphy scan at a median of 4 months after thrombectomy. A quantitative perfusion score was calculated for each planar ventilation/perfusion scintigraphy study to provide a % perfusion defect. Complete hemodynamic data were collected during the procedure and Miller score was calculated using prepulmonary and postpulmonary angiography. Echocardiographic data were collected prior to, 24 to 48 hours after, and 30 days after the procedure.

**Results:**

Four of 20 patients (20%) had ≥10% RPVO at a median of 4 months follow-up. Following MT, the mean Miller score decreased from 24.5 ± 2.9 to 15.8 ± 3.3 (*P* < .001) and mean pulmonary artery pressure decreased from 36.1 ± 4.8 mm Hg to 26.8 ± 5.4 mm Hg (*P* < .001). Right ventricle–to–left ventricle ratio decreased from 1.44 ± 0.2 to 1.05 ± 0.24 by 24 to 48 hours (*P* < .001) and 0.85 ± 0.1 at 30 days (*P* < .001) and right ventricular systolic pressure decreased from 63.2 ± 10 mm Hg to 42.1 ± 9.8 mm Hg at 24 to 48 hours (*P* < .001) and 31.9 ± 10.4 at 30 days (*P* < .001).

**Conclusions:**

In this prospective study of patients with submassive PE treated with MT, favorable rates of RPVO were noted in comparison to prior studies of anticoagulation alone along with expected acute hemodynamic and echocardiographic improvements. While this study was small in scope, the results suggest the potential for long-term benefits of MT in acute PE in addition to the acute benefits previously described.

## Central Illustration

**Figure:**
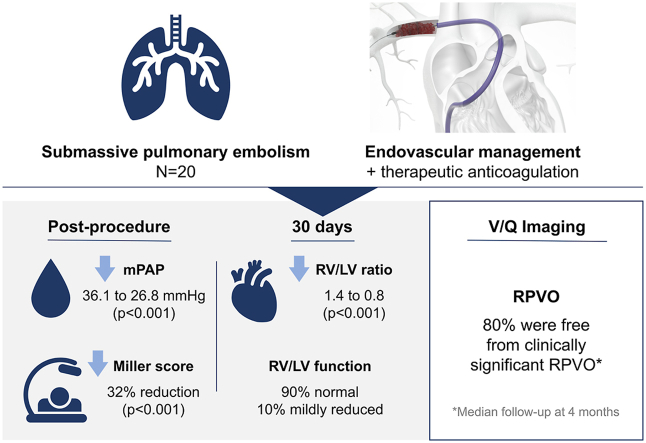

## Introduction

Pulmonary embolism (PE) is the third leading cause of cardiovascular death behind myocardial infarction and stroke.^1^ In patients who survive the initial embolic event, up to 50% suffer from some degree of morbidity.^2^ In the months following PE, residual pulmonary vascular obstruction (RPVO) observed by planar ventilation/perfusion scintigraphy (V/Q scan) has been associated with chronic dyspnea, recurrent venous thromboembolism (VTE), and chronic thromboembolic pulmonary hypertension.^3^^,^^4^ In previous studies of PE patients treated with anticoagulation alone, RPVO of >10% was seen in 46% to 59% of patients at 3 months and 25% to 29% at 1 year.^3^, ^4^, ^5^, ^6^, ^7^ Contemporary management of acute PE, particularly for submassive and massive PE, often involves endovascular intervention including percutaneous mechanical thrombectomy (MT). While this technology has demonstrated improvements in acute hemodynamics, oxygenation, and Right ventricle (RV)/left ventricle (LV) ratio, the long-term effects of these therapies relating to RPVO are poorly characterized.^8^^,^^9^

In this prospective, single-center study, we report acute outcomes and quantify subsequent RPVO by V/Q scan at follow-up in 20 consecutive submassive PE patients receiving MT with the FlowTriever system (Inari Medical).

## Methods

### Study design and population

This is a prospective, single-center, investigator-initiated, observational study of consecutive patients treated with the FlowTriever System for submassive PE who subsequently had planar V/Q imaging performed at a goal of 3 months follow-up. Sample size (n = 20) was determined a priori and, in total, 22 consecutive patients were enrolled with 20 completing the study. One patient declined follow-up V/Q scan due to COVID-19 concerns and 1 patient died prior to V/Q scan due to trauma unrelated to the PE. Institutional review board approval was obtained at the site and all patients provided written informed consent.

### Study device

The FlowTriever system consists of various aspiration catheters (16-24F) and optional nitinol disks. In treatment, the catheter is advanced through the right heart and into the pulmonary artery (PA) system. The nitinol disks can be deployed through the catheter to disrupt thrombus prior to aspiration.

### Pulmonary angiography

**Figure 1:**
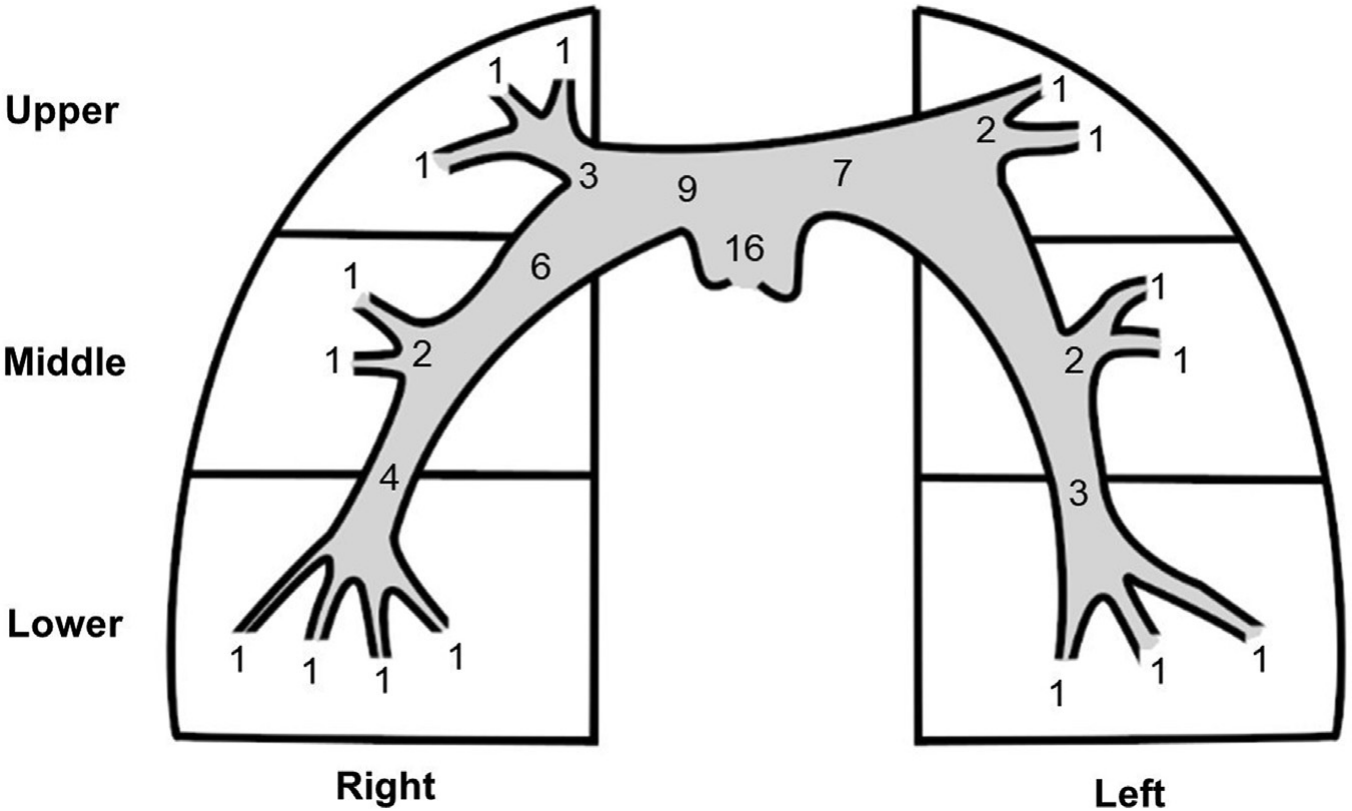
**Diagram of the components used to calculate the Miller score.** The arterial obstruction component is calculated from intravascular occlusion at each terminal vessel branch. The reduction in flow component is calculated for the upper, middle, and lower lobes of each lung (right, left). The image is redrawn from Miller et al.^10^

Pulmonary angiography was completed immediately before and after thrombectomy. There was no standard protocol for pulmonary angiography given multiple operators in an acute PE setting. Digital subtraction imaging was used in a majority of cases with use of a pigtail catheter and power injection for the baseline images with manual injection through the thrombectomy catheter for the postprocedural images. Images were later reviewed by a single reviewer (B.S.) and Miller score was calculated to quantify obstruction within the PAs. Miller score combines an objective measure of arterial obstruction with a semiobjective measure of the reduction in flow (Figure 1).^10^

1. To calculate the arterial obstruction component, the right and left PAs are divided into 9 and 7 major arterial branches, respectively. A filling defect present in any branch scores 1 point. Filling defects proximal to a group of branches are scored equal to the number of distal branches. Therefore, the maximum score for the arterial obstruction component is 16.
2. To calculate the reduction in flow component, the right and left lungs are divided into 3 perfusion zones (upper, middle, and lower). Flow in each zone is scored as absent (3 points), severely reduced (2 points), mildly reduced (1 point), or normal (0 points). Therefore, the maximum score for the reduction in flow component is 18.
3. Miller score is then calculated as the sum of the arterial obstruction and reduction in flow components divided by the maximum possible score of 34. Higher scores suggest greater obstruction of the PA tree and worse perfusion of the lungs.^10^

### Invasive hemodynamics and echocardiography

As per standard of care at our institution, all patients had PA pressures and mixed venous saturations obtained before and after thrombectomy with the latter allowing for calculation of Fick cardiac index.

Echocardiography was performed on the day of hospital admission prior to MT, at 24 to 48 hours after the procedure, and at 1 month after the procedure, as per standard of care at our institution. RV size and function were comprehensively evaluated in these imaging studies along with estimation of right ventricular systolic pressure when able to be obtained.

### V/Q imaging

Patients returned for a planar V/Q scan to evaluate RPVO at 3 months after procedure. Ventilation imaging was performed using aerosolized Tc-99m diethylenetriamine pentaacetate followed by perfusion imaging with Tc-99m macroaggregated albumin. Planar images were acquired in 8 standard views. No single-photon emission computed tomography or single-photon emission computed tomography/computed tomography imaging was performed. Images were analyzed by a single reviewer (D.A.).

For each patient, any significant perfusion defect that was considered consequent of PE was recorded. Significant perfusion defects were defined as medium-sized (>25% but <75% of a segment) or large (>75% of a segment), wedge-shaped, extending to the lung periphery, and not demonstrating a corresponding ventilation defect (“mismatched”). Small (<25% of a segment) perfusion defects were deemed insignificant and not recorded. All 18 standard pulmonary segments were evaluated in this manner and their size and location were recorded.

From this evaluation, RPVO was then calculated from modifications to a metric presented by Meyer et al^11^ as follows:

1. The lungs were divided into 5 lobes covering the 18 standard pulmonary segments: right superior lobe (segments 1-3), right middle lobe (segments 4-5), right inferior lobe (segments 6-10), left superior lobe (segments 11-14), and left inferior lobe (segments 15-18).
2. In each segment (1-18), a small perfusion defect (<25%) scored 0, a medium perfusion defect (>25% but <75%) scored 0.5, and a large perfusion defect (>75%) scored 1.
3. Each of the 5 lobes was prescribed a weight: right superior lobe (25%), right middle lobe (12%), right inferior lobe (18%), left superior lobe (25%), and left inferior lobe (20%). We assume that the segments contained within a lobe share equal proportions of the lobe’s total weight.
4. Lobar scores are then calculated by multiplying the segmental perfusion values by the predefined segmental weights and summed.
5. The lobar scores are then summed to determine the total perfusion score for a patient. RPVO is the complement to the total perfusion score (1–total perfusion score) and multiplied by 100 to provide a percentage of RPVO.
6. A minimum RPVO of 10% was applied. This threshold roughly corresponds to complete obstruction of at least 2 pulmonary segments and to the minimal high-probability result in diagnostic V/Q imaging^11^

### Statistical analysis

Categorical variables are reported as count (%) and mean ± SD and were compared using the χ^2^ test or Fisher exact test. Continuous variables are reported as median (IQR) and were compared using the 2-tailed *t* test or Mann–Whitney *U* test. The percentage change of a variable (eg, Miller score) was calculated as the change between time points divided by the initial time point and multiplied by 100. *P* values <.05 were considered significant. Analyses were performed using R version 3.6.3 Software (R Foundation for Statistical Computing). This study was approved by the institutional review board and written informed consent was provided.

## Results

### Patient characteristics

Baseline characteristics are presented in Table 1. The mean age was 63.3 ± 21.7 years, and mean body mass index was 35.85 ± 11.95 kg/m^2^. The majority of patients (55%, 11/20) were male. Rates of smoking (5%, 1/20), recent surgery (20%, 4/20), malignancy (10%, 2/20), and history of VTE (5%, 1/20), coronary artery disease (10%, 2/20), and congestive heart failure (5%, 1/20) were low. Troponin I levels were elevated in 18 patients (90%), and B-type natriuretic peptide levels were elevated in 11 patients (55%) but not evaluated in 3 patients.

**Table 1:** Baseline characteristics.

Characteristic	N = 20
Age, y	63.3 ± 21.7
Male sex	11 (55)
Body mass index, kg/m^2^	35.85 ± 11.95
Current smoker	1 (5)
History of deep vein thrombosis	1 (5)
History of pulmonary embolism	0 (0)
History of coronary artery disease	2 (10)
History of congestive heart failure	1 (5)
Recent surgery	4 (20)
Malignancy	2 (10)
Time to V/Q scan, min	123 (93-173.5)
Troponin I elevation	18 (90)
BNP elevation	11 (55)^a^

BNP, B-type natriuretic peptide; V/Q, planar ventilation/perfusion scintigraphy.

a B-type natriuretic peptide was not drawn in 3 patients.

### Pulmonary angiography and invasive hemodynamics

At the time of procedure, prepulmonary and postpulmonary angiography along with PA pressures and cardiac outputs were obtained as per standard of care at our institution. After thrombectomy, the mean Miller score decreased from 24.5 ± 2.9 mm Hg to 15.8 ± 3.3 mm Hg (*P* < .001), constituting an average 25.7% reduction in PA obstruction.

The mean PA pressure decreased from 36.1 ± 4.8 mm Hg to 26.8 ± 5.4 mm Hg (*P* < .001) and mean right atrial pressure decreased from 12.4 ± 3.6 mm Hg to 8.7 ± 4.9 mm Hg (*P* < .001). The Fick cardiac index was statistically unchanged before (1.79 ± 0.41 L/min/m^2^) and after procedure (1.69 ± 0.33 L/min/m^2^) (*P* = .25) (Table 2).

**Table 2:** Changes in invasive hemodynamic measurements.

Hemodynamic measurement	Before procedure (mean ± SD)	After procedure (mean ± SD)	Mean difference (% change)	*P* value
Mean pulmonary artery pressure, mm Hg	36.1 ± 4.8	26.8 ± 5.4	25.8	<.001
Mean right atrial pressure, mm Hg	12.3 ± 3.6	8.7 ± 4.9	29.2	<.001
Fick cardiac index, L/min/m^2^	1.79 ± 0.4	1.69 ± 0.3	5.6	.25
Heart rate, beats/min	91 ± 15.6	82.7 ± 17.9	9	<.001
Mean arterial pressure, mm Hg	107.3 ± 19.1	95.6 ± 16.4	10.9	<.001

### Echocardiography

**Figure 2:**
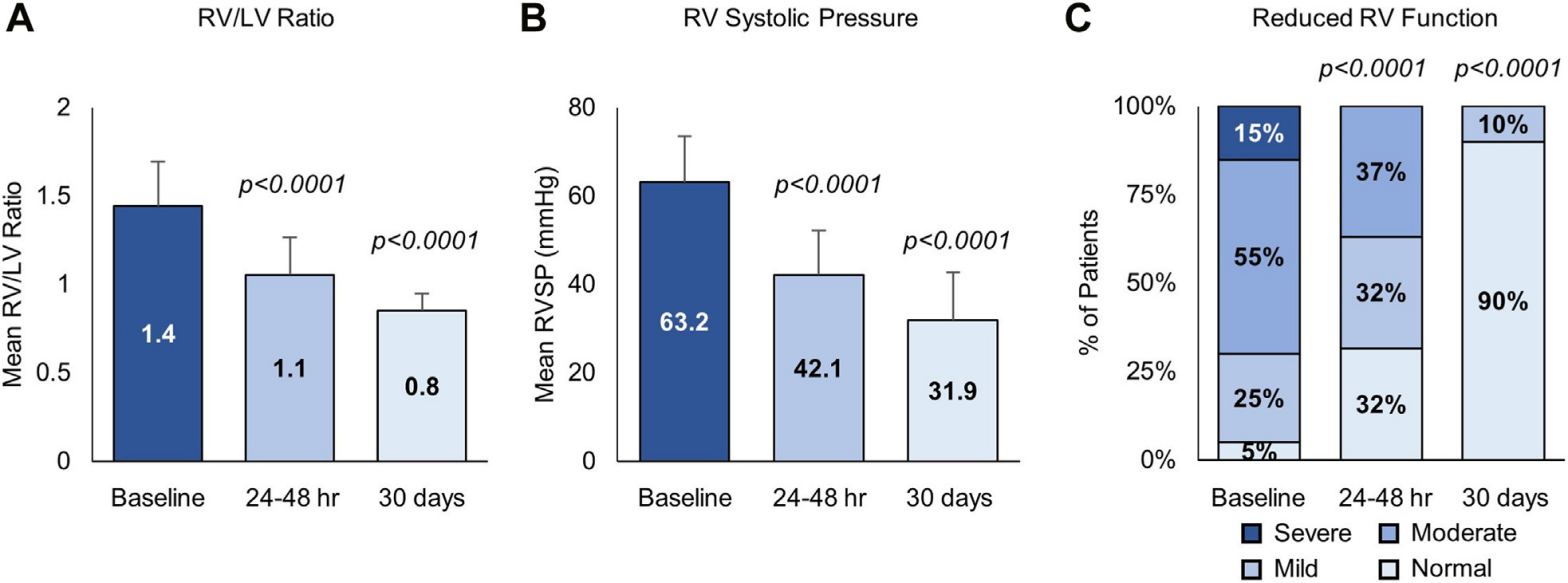
**Changes in echocardiographic measurements before, and 24 to 48 hours prior to thrombectomy.** (**A, B**) Report of the mean right ventricle (RV)/left ventricle (LV) ratio and right ventricular systolic pressure (RVSP) at each time point. Standard deviation is represented by whiskers. (**C**) The percentage of patients with reduced RV function, by category.

All patients had echocardiograms done at the time of presentation, at 24 to 48 hours after procedure, and at 1 month as per standard of care. RV/LV ratio decreased from 1.44 ± 0.2 at baseline to 1.05 ± 0.24 at 24 to 48 hours (*P* < .001) and 0.85 ± 0.1 at 30 days (*P* < .001). Right ventricular systolic pressure decreased from 63.2 ± 10 mm Hg at baseline to 42.1 ± 9.8 mm Hg at 24 to 48 hours (*P* < .001) and 31.9 ± 10.4 mm Hg at 30 days (*P* < .001). Both RV size and function were significantly improved at 24 to 48 hours and 30 days (Figure 2).

### Quantitative V/Q scan

**Figure 3:**
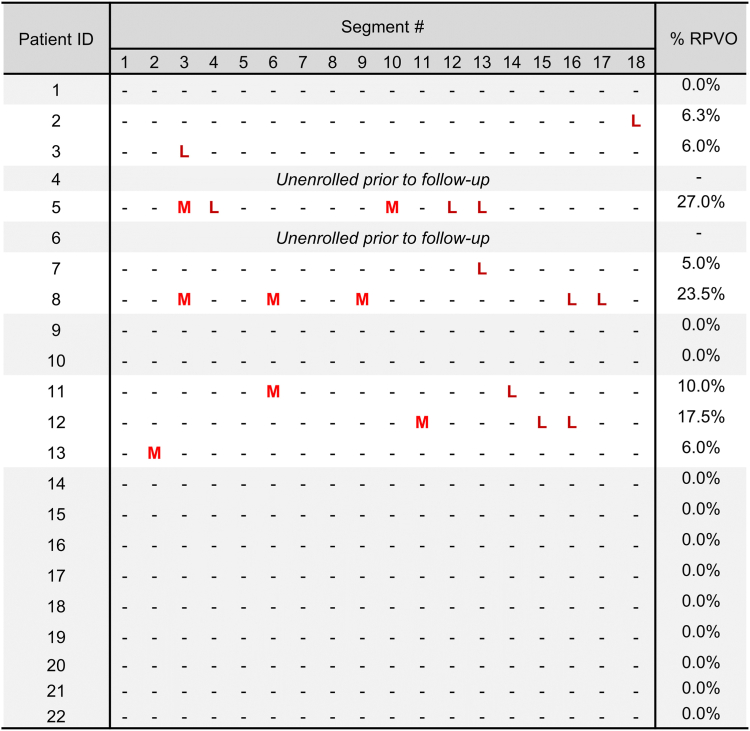
**Location and severity of residual pulmonary vascular obstruction (RPVO) from planar ventilation/perfusion scintigraphy imaging are shown for each subject.** L, large-sized perfusion defect (>75% of a segment); M, medium-sized perfusion defect (>25% but <75% of a segment).

The median time of V/Q scan administration after thrombectomy was at 4.0 months (IQR, 3.1-5.7 months). Variation in follow-up V/Q scan timing was largely related to scheduling complexities related to institutional COVID-19 policies. Figure 3 presents the location and size of perfusion defects and percentage of RPVO as quantified from V/Q imaging. The majority (80%, 16/20) of patients had either no discernible RPVO or limited RPVO (<10%), representing global perfusion mismatches of less than 10% at follow-up. The remaining 20% (4/20) of patients had RPVO ≥10%. In this study, no statistically significant association was noted between RPVO and hemodynamic and angiographic outcomes including reduction in Miller score, mean PA pressure, and RV/LV ratio.

## Discussion

This prospective, observational study of RPVO following MT in patients with submassive PE provides insight into potential for improvement in longer-term patient outcomes of which data are currently lacking. Reporting of RPVO is infrequent in modern literature, as V/Q imaging is cumbersome to acquire and rarely part of hospital standard of care. Although this study is small in scope, the acute results are comparable to larger registry data and the RPVO analysis contributes context to longer-term outcomes that have been reported (Central Illustration).

**Central Illustration:**
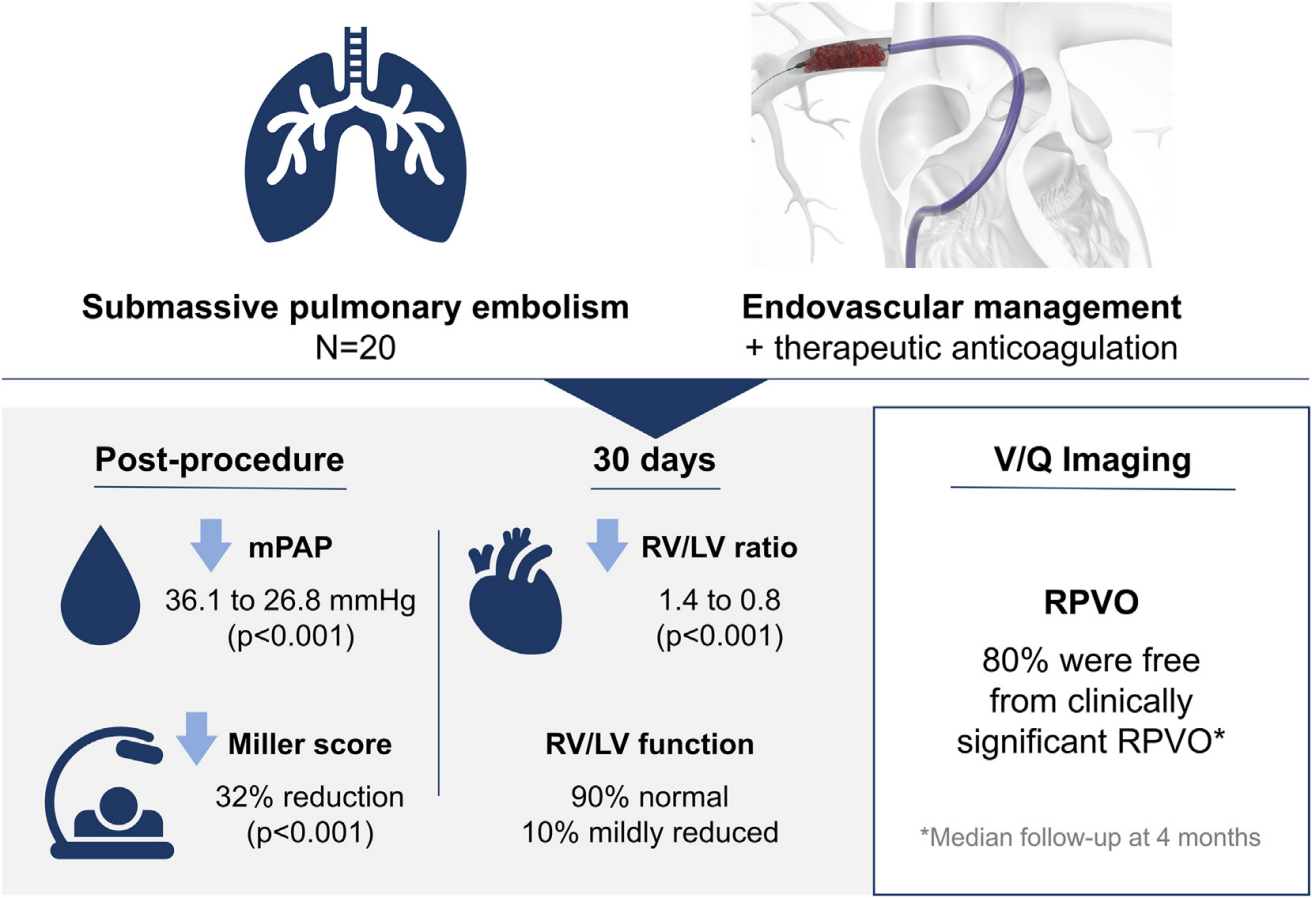
Summary of acute outcomes and longer-term residual pulmonary vascular obstruction after percutaneous thrombectomy in patients with submassive pulmonary embolism. LV, left ventricle; mPAP, mean pulmonary artery pressure; RPVO, residual pulmonary vascular obstruction; RV, right ventricle; V/Q, planar ventilation/perfusion scintigraphy.

Residual pulmonary vascular obstruction incidence at follow-up in this analysis (20%) compares favorably with previously published PE cohorts treated with anticoagulation alone over similar time periods (Table 3).^3^^,^^6^^,^^12^ These data offer potential evidence of the longer-term benefits of PE intervention with MT as RPVO has previously been associated with residual dyspnea, shorter 6-minute walk test, higher PA pressures, recurrent VTE, and greater incidence of chronic thromboembolic pulmonary hypertension.^3^^,^^4^

**Table 3:** Comparison of residual pulmonary vascular obstruction results to literature values with large sample sizes.

Reference, year	Median time to V/Q scan	Treatment	Imaging examination	N	% of patients with a defect	Average size of obstruction (%)
Sanchez et al,^3^ 2010	12 mo	AC, 6 m 6% had lytics	V/Q	254	29.0%	24.0 ± 16.0
Picart et al,^12^ 2020	6 mo	AC, mixed 1.7% had lytics	CT	537	35.6%	–
Wartski and Collignon,^6^ 2000	3 mo	AC, 3 m	V/Q	157	59%	19.0 ± 18.0
Current study	4 mo	FlowTriever	V/Q	20	20.0%	19.5 ± 7.0

AC, anticoagulation; CT, computed tomography; V/Q, planar ventilation/perfusion scintigraphy.

The mean reduction in Miller score (8.8 points) in this cohort is comparable to a recent study of MT using pulmonary angiography (7.5 points).^13^ Interestingly, mean Miller score was similar to numerically higher in this cohort (24.5) when compared to historical studies (range, 17-23), making it unlikely that the relatively low rate of clinically significant RPVO was related to a decreased baseline thrombus burden in this cohort, further suggesting treatment effect.^13^^,^^14^ Notably, pulmonary angiography was performed in an acute PE setting without a standard injection protocol potentially limiting the accuracy of comparisons with prior studies.

Herein, significant hemodynamic and echocardiographic improvements were observed at 48 hours which persisted to 30-day follow-up. These findings are consistent with acute outcomes reported from the FLASH registry.^9^ Although it is promising that these improvements reflect existing literature, the primary objective of this research was to expand clinical understanding by quantifying RPVO following MT, which is scarce in the literature.

Our study does include several limitations. Sample size was selected based on practical considerations as this was to be an exploratory, hypothesis-generating analysis that was not powered to allow for detection of any specific treatment effect. Given the small sample size, we are not able to adequately evaluate baseline predictors of residual RPVO or acute procedural outcomes that are associated with absence of significant long-term perfusion defects. This small sample size limits our ability to evaluate the potential association between RPVO and longer-term clinical outcomes in our population, though this has been established in prior studies.^2^, ^3^, ^4^ Lastly, RPVO was calculated using the above noted modifications to a metric presented by Meyer et al^11^ which may have varied slightly when compared to studies presented in Table 3.

## Conclusion

In this prospective study to quantify RPVO following MT for submassive PE, patients saw the expected acute echocardiographic and hemodynamic improvements following the procedure along with favorable rates of RPVO in comparison to prior studies of anticoagulation for PE. While this study was small in scope, we believe that results suggest there may be long-term benefits of MT in treating acute PE in addition to the acute benefits that have been previously described.
